# Functional Characteristics of Ultraviolet-Irradiated Tilapia Fish Skin Gelatin

**DOI:** 10.3390/molecules24020254

**Published:** 2019-01-11

**Authors:** Cheng-Kuo Wu, Jenn-Shou Tsai, Wen-Chieh Sung

**Affiliations:** Department of Food Science, National Taiwan Ocean University, 2 Pei-Ning Road, Keelung 20224, Taiwan; calvin26268@gmail.com (C.-K.W.); tsaijs@mail.ntou.edu.tw (J.-S.T.)

**Keywords:** gelatin, tilapia skin, gel strength, UV treatment

## Abstract

Studies were undertaken to investigate the effects of ultraviolet (UV) irradiation on the gel strength, color, thermal properties, protein molecular masses, and functional groups of commercially available fish gelatin samples. Commercially available tilapia skin gelatin powder was used as the raw material to investigate the functional properties of fish skin gelatin powder treated with UV irradiation for different durations (0–6 h). The functional properties of fish gelatin and the optimum irradiation treatment conditions were determined through gel strength testing, color characterization, differential scanning calorimetry, sodium dodecyl sulfate polyacrylamide gel electrophoresis, Fourier transform infrared (FTIR) spectroscopy, and Raman spectroscopy. UV irradiation treatment increased gel strength and thermal stability, and significantly degraded the macromolecules. FTIR and Raman spectroscopy data indicated that UV irradiation treatment did not significantly change the molecular structure of fish gelatin powder, but these methods could discriminate the molecular structure of gelatin from various sources. Irradiation for 2 h yielded the highest gel strength and melting peak temperature, and the lowest chromatic aberration.

## 1. Introduction

Gelatin, a partially hydrolyzed protein product derived from animal collagen, is soluble in hot water and forms a reversible gel widely used in the food, medicine, beauty, and photography industries [1,2,3].

Commercial gelatin is a raw material sourced from collagen and bone (or related byproducts) of pigs, cattle, and other animals. However, in recent years, animal diseases among cattle and pigs, such as mad cow disease (bovine spongiform encephalopathy, BSE) and foot and mouth disease (FMD), have brought into question the safety of the source materials used for gelatin [4,5]. During the Malaysia and Halal Food Marketing Strategy Forum in 2015, it was mentioned that there are 1.6 billion Muslim people across the world, accounting for nearly a quarter of the world’s population, and this number is expected to increase. The market demand for halal food is expected to increase as well. Muslim people follow strict religious rules and therefore, cannot eat pig-derived gelatin [6]. In addition, other countries, such as India and countries in East Asia that follow Buddhism, have also restricted the use of pig and cattle gelatin materials in food processing because of specific religious beliefs [2].

According to the 2015 data of the Fisheries Agency of the Executive Yuan Agricultural Committee [7], the tilapia production capacity in Taiwan is approximately 70,472 metric tons. Tilapia is primarily used in processed products, such as frozen fish fillets; most of the byproducts of processed foods were deemed feed waste in the past, thus leading to environmental pollution and the waste of resources. If more byproducts can be further extracted from this resource, for example, the use of fish skin extract to make the raw materials necessary to prepare fish gelatin—which would serve as an alternative to gelatin derived from terrestrial mammals—the value of processing aquatic products would increase and environmental pollution would decrease [8,9]. Therefore, fish gelatin has become a useful aquatic raw material. However, the rheological properties [10,11], gelation and melting temperatures, and gel strength [12] of fish gelatin are lower than that of mammalian gelatin because of the reduced hydroxyproline, proline, and imino acid content [2,13]. These properties limit fish gelatin’s application scope.

To improve the functional properties of fish gelatin, studies have examined whether adding various agents, such as sugars, salts, and glycerol, can modify the functional properties of fish gelatin [14,15]. However, adding such agents may affect subsequent processing recipes. Ultraviolet (UV) radiation technology is widely used in the sterilization of raw food materials and food contact surfaces. UV radiation technology uses non-free radiation and is a non-thermal processing technology, with such advantages as being cost effective and environmentally friendly, rendering it suitable for use in the food industry [16]. Exposing fish gelatin to UV radiation can significantly increase its gel strength, and this increase is proportional to the radiation dose [17,18]. Current research has focused on short-time UV-light-irradiated gelatin, with exposure times of 0 to 1 h [18,19], 0 to 1.5 h [17], and 0 to 3 h [20]; no long-term radiation treatment has yet been reported.

This study used commercially available tilapia fish skin gelatin powder as a raw material to investigate the effects of UV irradiation treatment on fish skin gelatin powder (UVFGP) for durations of 0 to 6 h and to determine the optimum processing conditions, with the objective of using UVFGP to replace mammalian gelatin. The functional properties of gelatin and the optimum irradiation treatment conditions were analyzed through gel strength analysis, colorimetry, differential scanning calorimetry (DSC), sodium dodecyl sulfate polyacrylamide gel electrophoresis (SDS-PAGE), Fourier transform infrared (FTIR) spectroscopy, and Raman spectroscopy.

## 2. Results and Discussion

### 2.1. Effect of Irradiation Dose on Gel Strength of Fish Gelatin Solution

The study examined the effect of various doses of UV irradiation on the gel strength of gelatin solution. Fish gelatin powder was treated for 0, 1, 2, 3, 4, 5, and 6 h, and the results are presented in Table 1. When the UV radiation dose was 1584 and 3168 mJ/cm^2^, the gel strength of the control and irradiated samples (218.9 ± 1.4 Bloom exposed for 1 h and 235.9 ± 1.7 Bloom exposed for 2 h) were significantly higher than that of the control sample (208.8 ± 1.5 Bloom). These results are consistent with those of Bhat and Karim [18], who also irradiated fish gelatin powder with UV light for 1 and 2 h. These findings indicate that the tilapia gelatin solution developed a relatively stable cross-linking structure on UV irradiation. The gel strength of tilapia gelatin gradually increased on irradiation for up to 2 h; however, no further increase in strength was noted when the irradiation time was extended to 3 h.

### 2.2. Effect of Irradiation Dose on the Color of Fish Gelatin Powder and Gel

Color is an essential indicator of raw food materials, and can be used to deduce the basic physical properties of irradiated gelatin powder. The changes in the color of gelatin powder after irradiation at different doses are presented in Table 2. The L* and b* values of the treated gelatin powder were all positive, and the a* value changed from negative to positive. The L* value decreased with increase in irradiation duration, reducing the brightness of the white gelatin powder. These findings indicate that high-molecular substances capable of absorbing visible light may be produced on irradiation [21]. The redness (a* value) and yellowness (b* value) increased with increase in irradiation duration, indicating that the primary protein structure of the gelatin had chemically changed, resulting in a slightly yellowish appearance. 

With increase in the dose of UV radiation, the color of the powder progressively changed from bright white to pinkish yellow. Chromatic aberration degree (ΔE) is a quantification of perceived difference in color and is calculated using L*, a*, and b* values; this parameter can be used to compare color differences between samples. When ΔE is 1.5–3.0, chromatic aberration is small yet noticeable, and when ΔE is 3.0–6.0, the aberration is significant [22]. On irradiation, ΔE of the irradiated samples increased compared with the control samples (Table 2); ΔE of tilapia gelatin powder was 1.85 and 2.79 after exposure for 1 and 2 h, respectively, indicating slight chromatic aberration and appreciable visual sensation, thus resulting in a relatively smaller and a significant sensation. However, the ΔE value was in the range of 3.63–5.01 after exposure for 2 h, indicating significant chromatic aberration and sensation.

**Figure 1:**
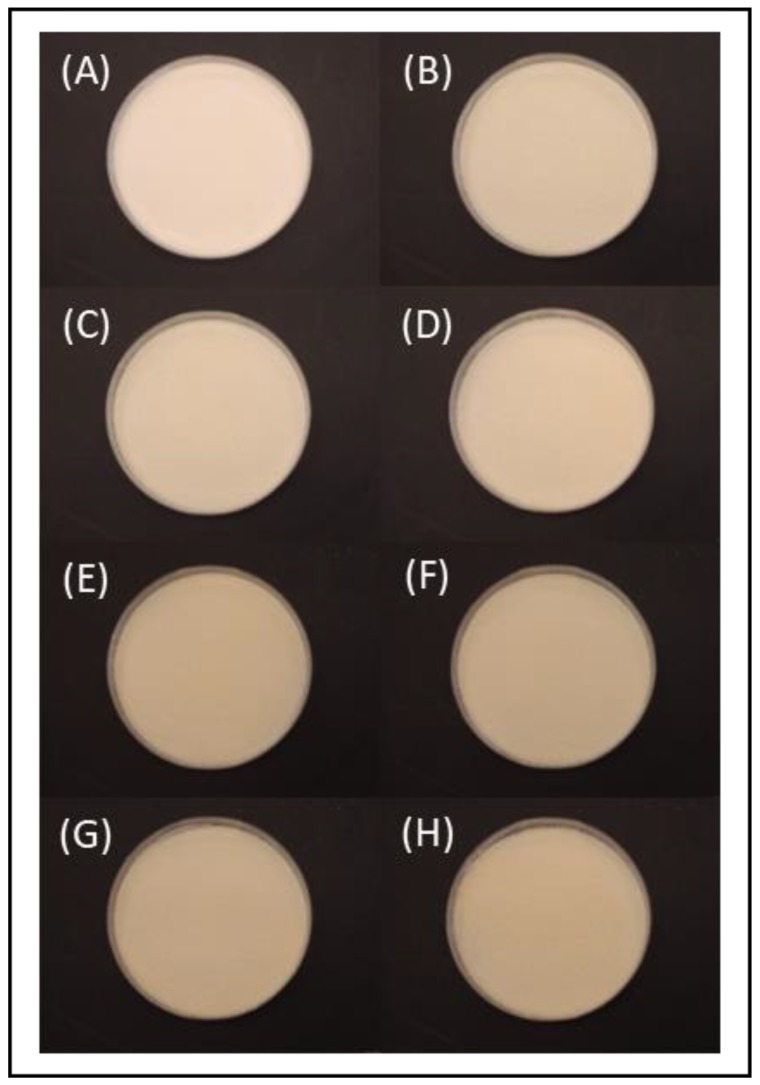
Photographs of (**A**) porcine gelatin gel and tilapia skin gelatin exposed to UV treatment for (**B**) 0 h; (**C**) 1 h; (**D**) 2 h; (**E**) 3 h; (**F**) 4 h; (**G**) 5 h; (**H**) 6 h.

Gelatin powder treated with different doses of UV irradiation was transformed into gels (Figure 1). The color of the gels noticeably darkened with increase in the UV dose, a result similar to that obtained for irradiated gelatin powder. Untreated fish gelatin gel was darker than porcine gelatin gel (Figure 1), and the difference in appearance between porcine gelatin and irradiated fish gelatin was more noticeable than that between porcine gelatin and untreated fish gelatin. This result differs from that of Chang et al. [20] because the present study used lower quantities of gelatin powder and a wider range of radiation doses.

### 2.3. Effect of Irradiation Dose on the Thermal Properties of Fish Gelatin Powder

The effect of UV irradiation dose (0, 1, 2, 3, 4, 5, and 6 h) on fish gelatin powder was examined through DSC; the results are presented in Table 3. Untreated fish gelatin powder had a melting peak temperature (Tp1) of 71.37 °C, and the 1 h treated fish gelatin sample had a Tp1 of 73.02 °C. Tp1 of the 2 h treated sample was 75.09 °C, an increase of 5.21% over the 1 h treated sample. The melting peak temperature and gel strength of the samples increased upon irradiation with UV light, indicating that upon UV radiation, the fish gelatin powder formed a relatively stable fish gelatin cross-linking structure. For samples irradiated for 3 to 6 h, Tp1 values of the samples slightly decreased and were between 71.32 °C and 72.08 °C, an insignificant increase compared with the 2 h treated sample.

Table 4 presents the results of the DSC analysis of 50 g porcine gelatin and 50 g fish gelatin powder exposed to UV irradiation. The melting peak temperature of porcine gelatin powder (74.19 °C) was greater than that of non-irradiated fish gelatin powder (71.37 °C), and the melting peak temperature of the 2 h treated sample (75.09 °C) was similar to that of commercially available porcine gelatin powder. Moreover, the melting peak temperature (Tp2) of porcine gelatin powder (221.91 °C) was higher than that of non-irradiated (217.58 °C) and irradiated fish gelatin powder (217.72 °C). The Tp2 values of the non-irradiated and irradiated samples did not differ significantly.

The results indicate that the UV-treated fish gelatin powder underwent radiation-induced chemical changes, and that the melting peak temperature of the powder can be increased by exposure for 2 h. The gel strength of the modified fish gelatin did not increase significantly when the irradiation duration exceeded 3 h.

### 2.4. Effect of Irradiation Dose on Gel Electrophoresis of Fish Gelatin Powder

**Figure 2:**
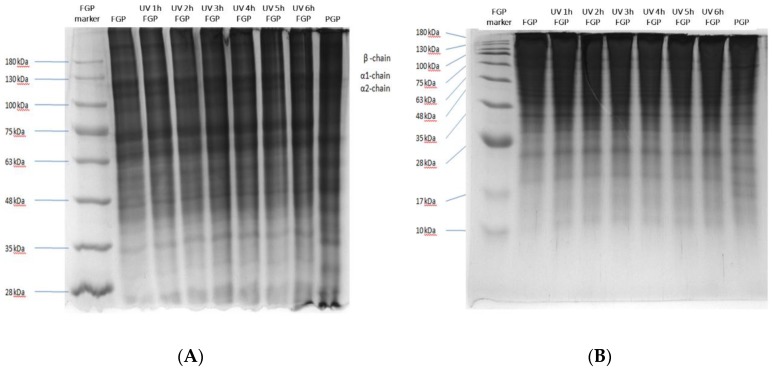
Sulfate polyacrylamide gel electrophoresis (SDS-PAGE) protein patterns of 50 g fish gelatin powder exposed to UV radiation. (**A**) Gel containing 8% acrylamide; (**B**) Gel containing 15% acrylamide. PGP = Porcine gelatin powder; FGP = Fish gelatin powder; UVFGP = UV-irradiated fish gelatin powder.

Figure 2 presents the SDS-PAGE patterns of gelatin powders, containing 8% and 15% separating gel (acrylamide), exposed to various irradiation doses. The pattern of the powder containing 8% acrylamide exhibited three bands—α1-chain, α2-chain, and 180 kDa β-component—at lower than the relative molecular weight of 130 kDa (Figure 2A). Increasing the UV radiation dose resulted in the blurring and widening of the three bands, and in the reduction of the color brightness relative to the non-irradiated powder. The protein molecular fragments of molecular weight lower than 63 kDa accumulated at the bottom of the gel electrophoresis pattern. The pattern also exhibited less noticeable changes when the powder was treated with low-energy UV radiation.

Similarly, the pattern of the irradiated sample containing 15% acrylamide exhibited a blurred and widened band and low color brightness relative to the non-irradiated fish gelatin powder at the relative molecular weight of 28 kDa (Figure 2B). These results indicate that on irradiation, the macromolecules in the fish gelatin powder slightly degraded with increase in the radiation dose, generating smaller molecular fragments than those accumulated at the bottom.

### 2.5. Effect of Irradiation Dose on FTIR of Fish Gelatin Powder

**Figure 3:**
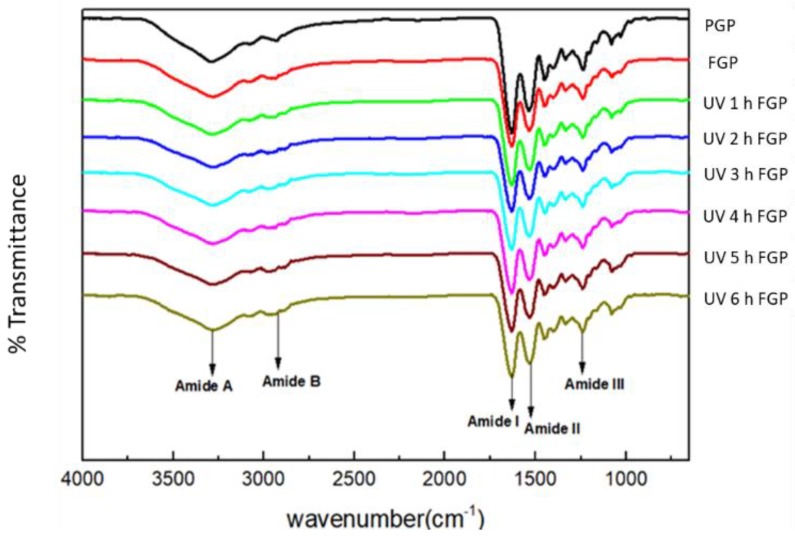
Fourier transform infrared (FTIR) spectra of 50 g fish gelatin powder and porcine gelatin powder exposed to UV radiation.

The effect of UV irradiation dose on the gelatin functional groups were examined through the FTIR spectrometry of fish gelatin powders exposed to 0, 1, 2, 3, 4, 5, and 6 h of irradiation (Figure 3). The spectral data indicated that the treated powders exhibited insignificant change in the amide A peak compared with the control group (FGP). The results for amide I, amide II, and amide III also showed insignificant change between control and experimental groups. However, when the peak was at the amide B, the fish gelatin powder experimental groups (UV 1 h–6 h FGP) treated with UV irradiation indicated a peak shape slightly different from that of the control group (FGP). These findings indicate that with increase in the dose of UV radiation, the amide B peaks slightly shifted in the FTIR spectra. The situation might be due to cross-linking that may occur in the gelatin granules after exposure to UV radiation. However, this inference will need to be proven through further investigation and research about the possible quantitative changes.

This result is similar to that of Bhat and Karim [19], but is different from that of Chang et al. [20]. The results of the present study indicate that fish gelatin powder treated with UV doses of 3600 to 21,600 mJ/cm^2^ undergoes a functional change in its molecular structure, namely a slight difference in the amide B group. The molecular structure of fish gelatin powder did not change significantly when the energy of the UV radiation energy was low.

Per the FTIR spectra in Figure 3, the porcine gelatin powder (PGP) and fish gelatin power (FGP) groups, but not the FGP and UV-irradiated fish gelatin powder (UVFGP) groups of porcine gelatin and fish gelatin powder irradiated for 2 h were significantly different. The gelatin powder from the PGP group was porcine, and the gelatin powders of FGP and UVFGP were from tilapia. This result therefore indicates that FTIR data can be used to distinguish between the molecular structures of gelatin powder from different sources.

### 2.6. Effect of Irradiation Dose on Raman Spectra of Fish Gelatin Powder

**Figure 4:**
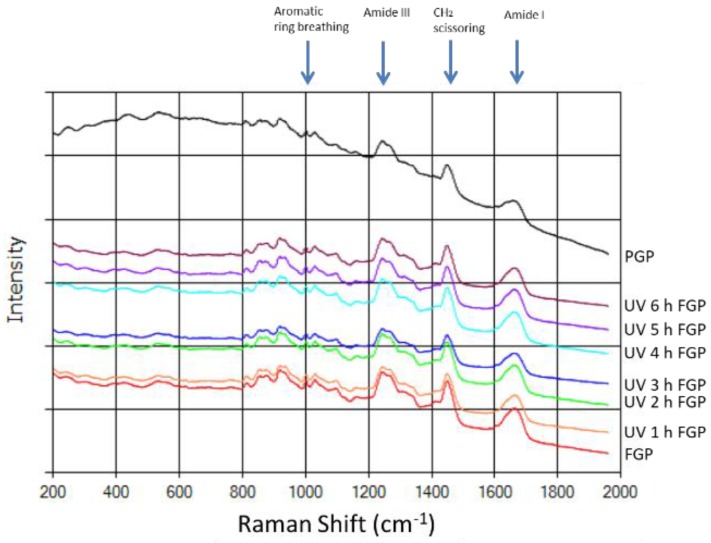
Raman spectra of porcine gelatin powder and 50 g of fish gelatin powder exposed to UV radiation.

Per the findings of Vandenabeele et al. [23] and other researchers on the functional group motions often observed in the Raman spectra of peptides, the amide I band is at 1670–1640 cm^−1^. The δ (CH_2_) deformations band is at 1480–1430 cm^−1^, the amide III band is at 1280–1100 cm^−1^, and the aromatic ring breathing band is at 1070–950 cm^−1^. In this study, fish gelatin powder was characterized through Raman spectra (Figure 4). The fish gelatin powder treated with UV irradiation exhibited a peak at the amide III band and the aromatic ring breathing band when irradiated for 0–6 h. No significant changes were observed in the control group. These findings indicate that the molecular structure of fish gelatin powder may not change significantly when the energy of the UV radiation is low.

Figure 4 also presents the Raman spectra of porcine gelatin, fish gelatin, and 50 g fish gelatin powder exposed to UV irradiation for 2 h. The percentage of aromatic ring structures in the porcine gelatin powder was higher than that of fish gelatin powder with or without irradiation treatment, and could be distinguished on the basis of the intensity ratio.

Table 5 presents three sets of intensity ratios. First, the intensity ratios (1450 cm^−1^/1412 cm^−1^) related to CH_2_ deformations indicate that the data obtained for the PGP and FGP groups differed significantly, whereas that for the UVFGP group did not. Second, per Sarbon et al. [24], the data obtained using this method revealed no significant difference, regardless of the group, when selecting the proline-related intensity ratio (1271 cm^−1^/1246 cm^−1^). Finally, the results indicate similarity with the aforementioned data by selecting the tyrosine-related intensity ratio (855 cm^−1^/830 cm^−1^).

The final results indicate that the data analysis of CH_2_ deformations and tyrosine in the PGP and FGP groups can be used to distinguish the groups, whereas the data obtained using proline cannot. However, the data of UVFGP obtained through this method did differ significantly. The Raman experiments confirmed that treating fish gelatin powder with low-energy UV irradiation did not significantly affect the molecular structure of the powder. Nevertheless, this method can be used to differentiate between the molecular structures of gelatin powder from different sources.

Yang and Wang [25] noted that the fibril structure was present in high concentrations (1%–6.67%) in gelatin, whereas spherical aggregates were present in low concentrations. Huang et al. [26] observed that untreated fish gelatin gel surface contained more voids than pectin and enzyme modified fish gelatin, indicating that the modified fish gelatin had improved gel strength and rheological properties.

## 3. Materials and Methods

### 3.1. Materials

Tilapia skin gelatin and porcine skin gelatin were purchased from the Jellice Pioneer Provate Limited Taiwan Branch (Pingtung, Taiwan). Each 100 g gelatin sample was sealed in a 1 kg polyethylene (PE) bag. All chemicals, namely acrylamide/bis-acryamide 30% solution, ammonia persulfate (APS), 0.5 M Tris-buffer (pH 6.8), 1.5 M tris-buffer (pH 8.8), sodium dodecyl sulfate (SDS), and N, N, N0, N0-tetramethylethylenediamine (TEMED), for the electrophoresis were purchased from Bio Basic Inc. (Toronto, ON, Canada).

### 3.2. Tilapia Gelatin Powder Pretreatment

This study adopted the method used by Sung and Chen [27], with few modifications. Fish gelatin powder (50 g) was placed on a stainless sheet plate (21 × 29 cm) with a powder thickness of nearly 1.5 mm. The UV light C tube (Model Allkill-01, 253.7 nm; 30W, PJLink, Taipei, Taiwan) was used as the UV light source, and the lamp tube clamp was fixed on a steel frame; the irradiation distance was adjusted to 30 cm for the UV irradiation treatment. The UV light irradiation pretreatment equipment of the fish skin gelatin was placed on a table in an aseptic operation room, and the UV irradiation durations were 1, 2, 3, 4, 5, and 6 h. All experimental treatments were conducted in triplicate. The irradiated samples were sealed into PE bags at room temperature (25 °C) until subsequent analyses.

### 3.3. UV Irradiance Measurement

UV radiation dose measurements were performed per manufacturer instructions. Using a UV light meter (ST512, Sentry Industries Inc., Hillburn, NY, USA) with a spectral range of 220–275 nm, illuminance (mw/cm^2^) measurements were performed using UV light at nine points across the stainless sheet plate and averaged. Table 6 presents the UV irradiation exposure durations and corresponding UV radiation doses. The UV irradiation dose per unit area can be calculated by multiplying the average irradiance with the continuous irradiation duration, per the formula used by Craik et al. [28]:

UVR dose (mJ/cm^2^) = Effective Average Irradiance (mw/cm^2^) × Exposure time (s) (1)

### 3.4. Gel Strength of Gelatin Solution

This study followed the method developed by the British Standard Institute in 1975 [29]. The gelatin powder was dissolved in distilled water at 60 °C to obtain a 6.67% (*w*/*v*) colloidal solution; it was then poured into a test bottle (3.8 cm diameter × 2.7 cm high). The prepared solution was placed in a 10 °C refrigerator for cooling and kept for 16–18 h. After completing the sample preparation, it was immediately removed for testing of its gel strength using a texture analyzer (TA.XT2, Stable Micro Systems, Godalming, Surrey, UK) and a P/0.5 cylindrical probe of diameter 1.27 cm. The test speed was 0.5 mm/s and the penetration distance was 4 mm. These gel strength measurements were performed by analyzing the force–time curve of the gelatin solution in triplicate.

### 3.5. Color Measurement of Gelatin Powder

The CIELAB color (L*, a*, b*) and total color difference (ΔE) of tilapia skin gelatin powder was measured using a colorimeter (Chroma meter CR-410, Konica Minolta, Japan). After using a whiteboard for calibration, the fish gelatin powder was placed in a scanning spectrophotometer sample tray, followed by L*, a*, and b* determination. L* values range from 0 to 100 and represent the lightness of the color; L* = 0 represents black and L* = 100 represents white. Values for a* and b* range from −60 to 60, with negative and positive a* signifying green and red, respectively, and negative and positive b* signifying blue and yellow, respectively [30]. All tests were performed in triplicate. The measured L*, a*, and b* values were substituted into the following formula to calculate the total color difference (ΔE) [31].

ΔE = [(ΔL*)^2^ + (Δa*)^2^ + (Δb*)^2^]^1/2^ (2)

ΔL = L*_sample_ − L*_control_; Δa= a* _sample_ − a* _control_; Δb = b* _sample_ − b* _control_ (3)

### 3.6. DSC Analysis of Gelatin Powder

The DSC (Mettler, Toledo, Switzerland) analysis of gelatin powder was conducted using a method adopted from Rahman et al. [32], but with slight modifications. The sample (5 mg) was placed in a dedicated analytical 40 μL standard aluminum crucible, sealed with the lid of a standard aluminum crucible and weighted. The samples were scanned from −60 °C to 250 °C at a heating rate of 20 K/min; the reaction gas was N_2_, which was released at 50 mL/min. The thermal stability analysis of the gelatin powder was performed in triplicate to record the change of heat enthalpy (ΔH) during the test; the onset temperature (To), peak temperature (Tp), and enthalpy data values were also recorded.

### 3.7. SDS-PAGE Analysis of Gelatin

This study adopted a method reported by Lin et al. [33]. The gel solution was composed of acrylamide (running gel with 8% and 15% (*v*/*v*)) and stacking gel (4% (*v*/*v*) of acrylamide). After the gel solidified, 20 μg of protein was pipetted into each well, and a voltage of 60 volts was applied. Subsequently, the gel was stained using Coomassie brilliant blue R250, and the molecular weight was determined using a molecular-weight size marker. The molecular size was relatively small because it was a fast protein.

### 3.8. FTIR Spectral Analysis of Gelatin Powder

This study adopted the method reported by Benjakul et al. [34]. Fish gelatin powder and potassium bromide were uniformly mixed at a ratio of 1:100 and dried in an oven at 50 °C for 24 h. The mixed powder was then removed, transformed into pellets using a hydraulic press machine, and subjected to FTIR spectrometry (FT-IR, Perkin Elmer, Spectrum One, San Diego, CA, USA) at 25 °C ± 2 °C and a scan resolution of 4 cm^−1^; scanning was performed 32 times. The spectral wave number was in the IR range of 650–4000 cm^−1^.

### 3.9. Raman Spectroscopy Analysis of Gelatin Powder

This study adopted a version of the method reported by Sarbon et al. [24]. This analysis did not require pretreatment of the fish gelatin powder samples, which were directly characterized using a Raman spectrometer (Acuscan 1500, Acutech scientific Inc., San Diego, CA, USA). The wave number range was 200–2000 cm^−1^, and a 785 nm laser was used as the excitation source. The samples were analyzed at a laser power of 100 mW, and all assays were performed in triplicate.

### 3.10. Statistical Analysis

All experiments were repeated at least thrice. The results were analyzed using one-way analysis of variance (ANOVA), and Duncan’s new multirange tests by using SPSS (Version 12.0, SPSS, 2000, Chicago, IL, USA) with the significance level set at *p* < 0.05.

## 4. Conclusions

According to the gel strength and color analysis, the color of the fish gelatin powder gradually changed from bright white to slightly yellowish after irradiation with UV light. The gel strength and the melting peak temperature of gelatin increased significantly and was the highest when irradiated for 2 h; the color underwent noticeable chromatic aberration. When the samples were irradiated for more than 2 h, the increase in gel strength and melting peak temperature was insignificant and was accompanied by a substantial yellowing phenomenon. Thus, irradiation for 2 h is optimal.

The peak temperature of melting and cracking, as determined through DSC analysis, indicated that fish skin gelatin has poor thermal stability compared with commercially available porcine gelatin powder. Nevertheless, fish gelatin powder modified through optimal UV irradiation exhibited improved melting peak temperature and thermal stability similar to that of porcine gelatin powder, but the cracking peak temperature did not change significantly.

Gel electrophoresis and optical spectral analyses revealed that with increased dose of UV radiation, the macromolecules of the gelatin powder degraded and accumulated at the bottom. The FTIR and Raman spectra also confirmed this finding. Thus, FTIR and Raman analyses can be used to differentiate between gelatin powders from different sources.

## Figures and Tables

**Table 1:** Effect of UV irradiation on the gel strength of fish gelatin powder.

Exposure Time (h)	UVR Dose * (mJ/cm^2^)	Gel Strength ** (Bloom)
0	0	208.8 ± 1.5 d
1	1584	218.9 ± 1.4 c
2	3168	235.9 ± 1.7 a
3	4752	222.3 ± 2.0 b
4	6336	221.8 ± 1.9 bc
5	7920	223.3 ± 2.6 bc
6	9504	224.6 ± 2.1 b

* UVR dose = UV radiation dose; ** Gel strength = Gelatin strength (mean ± SD); entries with different letters (a–d) differ significantly (*p* < 0.05), *n* = 3.

**Table 2:** Effect of UV radiation on the coloration of fish gelatin powder.

Exposure Time (h)	UVR Dose (mJ/cm^2^)	Color of Fish Gelatin Powder			
		L* Value	a* Value	b* Value	ΔE
0	0	88.81 ± 0.07 a	−2.78 ± 0.16 e	14.74 ± 0.14 c	Control
1	1584	88.11 ± 0.36 b	−1.11 ± 0.26 d	14.96 ± 0.38 c	1.85 ± 0.32 e
2	3168	87.54 ± 0.10 c	−0.51 ± 0.55 d	15.71 ± 0.34 b	2.79 ± 0.48 d
3	4752	87.41 ± 0.01 c	0.25 ± 0.13 c	16.15 ± 0.11 ab	3.63 ± 0.15 c
4	6336	87.28 ± 0.10 cd	0.55 ± 0.40 bc	16.22 ± 0.55 ab	3.97 ± 0.43 bc
5	7920	87.02 ± 0.05 de	1.00 ± 0.59 ab	16.51 ± 0.30 a	4.56 ± 0.62 ab
6	9504	86.92 ± 0.05 e	1.46 ± 0.41 a	16.58 ± 0.22 a	5.01 ± 0.27 a

Note: UVR dose = UV radiation dose. Values are presented as mean ± SD from triplicate measurements. Different letters (a–e) in the same column differ significantly (*p* < 0.05). ΔE = [(ΔL)^2^ + (Δa)^2^ + (Δb)^2^]^1/2^; ΔL = L*sample − L*control; Δa = a* sample − a* control; Δb = b* sample − b* control.

**Table 3:** Differential scanning calorimetry (DSC) of UV-irradiated fish gelatin powder.

Sample	UVR Dose (mJ/cm^2^)	To1 (°C)	Tp1 (°C)	Normalized1 (J/g)	To2 (°C)	Tp2 (°C)	Normalized2 (J/g)
FGP	0	62.99 ± 0.41 ^b^	71.37 ± 0.21 ^c^	−34.20 ± 3.16 ^ab^	209.52 ± 0.46 ^c^	217.58 ± 0.23 ^a^	−14.92 ± 3.47 ^bc^
UV 1h FGP	1584	65.06 ± 2.08 ^b^	73.02 ± 1.41 ^b^	−37.02 ± 5.45 ^ab^	211.49 ± 0.64 ^a^	218.01 ± 0.41 ^a^	−9.02 ± 1.40 ^a^
UV 2h FGP	3168	68.22 ± 0.94 ^a^	75.09 ± 0.67 ^a^	−33.80 ± 3.43 ^ab^	210.66 ± 0.18 ^b^	217.72 ± 0.24 ^a^	−13.55 ± 0.88 ^ab^
UV 3h FGP	4752	63.68 ± 1.71 ^b^	71.92 ± 0.68 ^bc^	−39.78 ± 8.73 ^ab^	209.58 ± 0.32 ^c^	217.61 ± 0.37 ^a^	−15.37 ± 3.97 ^bc^
UV 4h FGP	6336	64.37 ± 0.67 ^b^	72.08 ± 0.86 ^bc^	−37.89 ± 2.81 ^ab^	209.82 ± 0.39 ^c^	217.77 ± 0.04 ^a^	−13.22 ± 1.83 ^ab^
UV 5h FGP	7920	63.60 ± 1.09 ^b^	71.32 ± 0.88 ^c^	−43.52 ± 0.68 ^b^	209.26 ± 0.35 ^c^	217.67 ± 0.17 ^a^	−19.80 ± 0.71 ^c^
UV 6h FGP	9504	62.41 ± 2.49 ^b^	72.04 ± 0.13 ^bc^	−30.74 ± 6.59 ^a^	209.39 ± 0.53 ^c^	217.77 ± 0.11 ^a^	−19.45 ± 4.53 ^c^

Note: FGP = fish gelatin powder; UVFGP = UV-irradiated fish gelatin powder; UVR dose = UV radiation dose; To1 = onset of melting temperature; Tp1 = peak of melting temperature; Normalized1 = enthalpy (ΔH_1_) of melting; To2 = onset of pyrolysis temperature; Tp2 = peak of pyrolysis temperature; Normalized2 = enthalpy of pyrolysis (ΔH_2_); mean ± SD within the same column, values with different superscripts differ significantly (*p* < 0.05), *n* = 3.

**Table 4:** DSC of porcine gelatin powder, fish gelatin powder, and 50 g fish gelatin powder exposed to UV radiation.

Sample UVR Dose	(mJ/cm^2^)	PGP	FGP	UV 2 h FGP
		0	0	3168
To1	(°C)	66.86 ± 1.10 ^a^	62.99 ± 0.41 ^b^	68.22 ± 0.94 ^a^
Tp1	(°C)	74.19 ± 1.61 ^a^	71.37 ± 0.21 ^b^	75.09 ± 0.67 ^a^
Normalized1	(Jg^−1^)	−39.33 ± 10.87 ^a^	−34.20 ± 3.16 ^a^	−33.80 ± 3.43 ^a^
To2	(°C)	212.95 ± 2.04 ^a^	209.52 ± 0.46 ^b^	210.66 ± 0.18 ^ab^
Tp2	(°C)	221.91 ± 0.93 ^a^	217.58 ± 0.23 ^b^	217.72 ± 0.24 ^b^
Normalized2	(Jg^−1^)	−59.53 ± 3.97 ^b^	−14.92 ± 3.47 ^a^	−13.55 ± 0.88 ^a^

Note: PGP = Porcine gelatin powder; FGP = Fish gelatin powder; UVFGP = UV-irradiated fish gelatin powder; UVR dose = UV radiation dose; To1 = onset of melting temperature; Tp1 = peak of melting temperature; Normalized1 = enthalpy (ΔH_1_) of melting; To2 = onset of pyrolysis temperature; Tp2 = peak of pyrolysis temperature; Normalized2 = enthalpy of pyrolysis (ΔH_2_); Mean ± SD within the same column, values with different superscripts differ significantly (*p* < 0.05), *n* = 3.

**Table 5:** Change in ratio I_1450_/I_1412_, I_1271_/I_1246,_ and I_855_/I_830_ in the Raman spectroscopic analysis of porcine gelatin powder and UV-irradiated 50 g fish gelatin powder.

Sample	Intensity		Ratio: CH_2_ Deformations	Intensity		Ratio: Proline	Intensity		Ratio: Tyrosine
	Raman Shift		Ratio	Raman Shift		Ratio	Raman Shift		Ratio
	1450 cm^−1^	1412 cm^−1^	1450/1412	1271 cm^−1^	1246 cm^−1^	1271/1246	855 cm^−1^	830 cm^−1^	855/830
PGP	25,655	23,373	1.10	29,190	30,352	0.96	34,465	33,510	1.03
FGP	17,881	13,806	1.30	18,834	19,657	0.96	18,801	16,767	1.12
UV1hFGP	14,369	10,786	1.33	15,014	15,672	0.96	15,161	13,148	1.15
UV2hFGP	15,606	11,977	1.30	16,355	17,098	0.96	16,516	14,595	1.13
UV3hFGP	11,947	9239	1.29	12,581	13,159	0.96	12,926	11,471	1.13
UV4hFGP	16,358	12,657	1.29	17,283	18,050	0.96	17,676	15,654	1.13
UV5hFGP	15,557	11,894	1.31	16,254	17,062	0.95	16,626	14,420	1.15
UV6hFGP	14,681	11,073	1.33	15,225	16,026	0.95	14,974	13,217	1.13

Note: Condition: Laser wavelength 785 nm. Laser Power 100 mW. Dark noise subtraction mode. PGP = Porcine gelatin powder; FGP = Fish gelatin powder; UVFGP = UV-irradiated fish gelatin powder.

**Table 6:** UV radiation exposure durations for their relative UV radiation doses.

Exposure Time (h)	Exposure Time (s)	UVR Dose ^a^ (mJ/cm^2^)
0	0	0
1	3600	1584
2	7200	3168
3	10,800	4752
4	14,400	6336
5	18,000	7920
6	21,600	9504

Note: ^a^ UVR dose = UV radiation dose.
